# Transcriptome profiling in imipenem-selected *Acinetobacter baumannii*

**DOI:** 10.1186/1471-2164-15-815

**Published:** 2014-09-26

**Authors:** Kai-Chih Chang, Han-Yueh Kuo, Chuan Yi Tang, Cheng-Wei Chang, Chia-Wei Lu, Chih-Chin Liu, Huei-Ru Lin, Kuan-Hsueh Chen, Ming-Li Liou

**Affiliations:** Department of Laboratory Medicine and Biotechnology, Tzu Chi University, Hualien City, Taiwan; Department of Medicine, National Taiwan University Hospital Hsin-Chu Branch, Hsin-Chu City, Taiwan; College of Medicine, National Taiwan University, Taipei City, Taiwan; Department of Computer Science and Information Engineering, Providence University, Taichung, Taichung County Taiwan; Department of Computer Science, National Tsing Hua University, Hsin-Chu City, Taiwan; Department of Bioinformatics, Chung Hua University, Hsin-Chu City, Taiwan; Department of Medical Laboratory Science and Biotechnology, Yuanpei University, No. 306, Yuanpei Street, Hsin-Chu, 30015 Taiwan

**Keywords:** *Acinetobacter baumannii*, Carbapenem resistance, Transcriptome profiling

## Abstract

**Background:**

Carbapenem-resistance in *Acinetobacter baumannii* has gradually become a global challenge. To identify the genes involved in carbapenem resistance in *A. baumannii*, the transcriptomic responses of the completely sequenced strain ATCC 17978 selected with 0.5 mg/L (IPM-2 m) and 2 mg/L (IPM-8 m) imipenem were investigated using RNA-sequencing to identify differences in the gene expression patterns.

**Results:**

A total of 88 and 68 genes were differentially expressed in response to IPM-2 m and IPM-8 m selection, respectively. Among the expressed genes, 50 genes were highly expressed in IPM-2 m, 30 genes were highly expressed in IPM-8 m, and 38 genes were expressed common in both strains. Six groups of genes were simultaneously expressed in IPM-2 m and IPM-8 m mutants. The three gene groups involved in DNA recombination were up-regulated, including recombinase, transposase and DNA repair, and beta-lactamase OXA-95 and homologous recombination. The remaining gene groups involved in biofilm formation were down-regulated, including quorum sensing, secretion systems, and the csu operon. The antibiotic resistance determinants, including RND efflux transporters and multidrug resistance pumps, were over-expressed in response to IPM-2 m selection, followed by a decrease in response to IPM-8 m selection. Among the genes over-expressed in both strains, *bla*_OXA-95,_ previously clustered with the *bla*_OXA-51-like_ family, showed 14-fold (IPM-2 m) to 330-fold (IPM-8 m) over-expression. The expression of *bla*_OXA-95_ in IPM-2 m and IPM-8 m cells was positively correlated with the rate of imipenem hydrolysis, as demonstrated through Liquid Chromatography-Mass Spectrometry/Mass Spectrometry, suggesting that *bla*_OXA-95_ plays a critical role in conferring carbapenem resistance. In addition, *A. baumannii* shows an inverse relationship between carbapenem resistance and biofilm production.

**Conclusion:**

Gene recombination and *bla*_OXA-95_ play critical roles in carbapenem resistance in *A. baumannii*. Taken together, the results of the present study provide a foundation for future studies of the network systems associated with carbapenem resistance.

**Electronic supplementary material:**

The online version of this article (doi:10.1186/1471-2164-15-815) contains supplementary material, which is available to authorized users.

## Background

In the last decades, *A. baumannii* has gradually emerged as an important nosocomial pathogen worldwide, reflecting antimicrobial resistance, tolerance to desiccation and disinfection and biofilm formation on common abiotic surfaces in healthcare settings [1]. Carbapenems, primarily imipenem and meropenem, have been used to treat multidrug-resistant (MDR) *A. baumannii* infections [1]. However, the increasing incidence of carbapenem-resistant *A. baumannii* (CRAB) infections in Taiwan and many other countries has become of critical concern [2].

Currently, several MDR determinants contribute to the antimicrobial resistance observed in this microorganism. The most prevalent MDR determinants in *A. baumannii* include genes for efflux pumps, class B β-lactamase (metallo-beta-lactamase), class C chromosomal β-lactamase AmpC, class D β-lactamase (OXA-type carbapenemase), integrons and associated insertion sequence (IS) elements [1]. Virulence factors associated with resistance, including biofilm formation, and surface and extracellular polysaccharides associated with capsule formation have also been demonstrated [3]. While progress has been made in characterizing the determinants of antibiotic resistance in this organism, few reports have shown the expression patterns or mechanisms underlying the acquisition or control of these genes.

To characterize the antimicrobial resistance mechanisms underlying MDR in *A. baumannii*, several approaches to examine gene expression profiles have been developed. Proteomics methods using two-dimensional electrophoresis (2D) and Liquid Chromatography-Mass Spectrometry/Mass Spectrometry (LC-MS/MS) have been used to examine changes in *A. baumannii* protein expression associated with drug resistance [4–6]. Yun *et al*. [4] identified 484 proteins with expression differences in the clinical MDR strain DU202 subjected to sub-minimum inhibitory concentration levels of tetracycline and imipenem. Chopra *et al*. [5] compared the proteome of the MDR strain BAA-1605 and a reference strain, identifying nearly 200 proteins with expression differences between the two strains. Indeed, the proteomics approaches using 2D and LC-MS/MS might provide large-scale proteomics involved in antibiotic resistance; however, less than 25% of proteins could be detected, reflecting the limitations of 2D approaches. Also, microarray technology has been used to screen and quantify the expression profiles of antibiotic resistance genes in *A. baumannii* [7]. However, this approach has been restricted to the study of previously known genes, which would not reveal the entire transcriptional profile of genes expressed upon exposure to antibiotics.

Among the recent techniques used to analyze the whole RNA profiles of microorganisms are next generation sequencing (NGS), 454 GS_FLX (Roche), MiSeq or HiSeq (Illumina Inc.) platforms, and ABI SOLiD (Life Technology). RNA sequencing using the Illumina system has been regarded as an extremely informative technique for the study of transcriptional profiles of microorganisms, as these techniques are sensitive and rapid [8, 9]. However, in the last two years, there have only very few studies using RNA-sequencing technologies in *A. baumannii* [10–12]. Using transcriptional profiling and functional assays in a mutant strain, Cerqueira *et al*. [12] identified a global virulence regulator in *A. baumannii* that controls the phenylactic acid catabolic pathway. Using the same approach, Eijkelkamp *et al*. [10] also identified a role for the gene encoding a homolog of the histone-like nucleoid structuring (H-NS) protein involved in *A. baumannii* virulence. Currently, there is only one report concerning the whole transcriptome analysis of the genes involved in biofilm formation in *A. baumannii* [11]. Rumbo-Feal *et al*. [11] identified 1621 genes over-expressed in biofilms relative to stationary phase cells and 55 genes expressed only in biofilms. Among the genes over-expressed in biofilms were those involved in quorum sensing and the CsuAB-A-B-C-D-E chaperone-usher secretion system. Although biofilm formation has been implicated in antibiotic resistance in bacteria [13], the correlation between antibiotic resistance and biofilm formation in *A. baumannii* remains poorly understood.

In a previous study [14], we employed genome-wide analysis to characterize the potential resistance mechanisms in *Acinetobacter baumannii* ATCC 17978 following imipenem exposure. Genome-wide analysis showed that exposure to 0.5 mg/L imipenem mediated the transposition of IS*Aba1*, located upstream of the *bla*_OXA-95_ gene, resulting in the overexpression of the *bla*_OXA-95_ gene. Thus, the aim of the present study was to investigate the carbapenem resistance mechanism in *A. baumannii* using the Illumina RNA-sequencing technologies. We therefore obtained transcriptome profiles from *A. baumannii* ATCC 17978 and its carbapenem-selected mutants, and these profiles were compared to identify differences in the gene expression profiles. The results of the present study will provide insight into the mechanisms underlying carbapenem resistance and their association with biofilm formation in *A. baumannii*.

## Results

### Susceptibility testing

Antibiotic-selected mutants were generated from the ATCC 17978 type strain. The identities of the selected mutants originated from ATCC 17978 were confirmed using pulsed-field gel electrophoresis (PFGE). The results of antibiotic susceptibility testing for these mutants and the parental strain are shown in Table 1. The reference strain 17978 was susceptible to all antibiotics tested. The MICs to meropenem and imipenem were increased more than four-fold in response to IPM-2 m and IPM-8 m selection at concentrations of 0.5 and 2 mg/L imipenem. In addition, the MICs of the imipenem-selected mutants to the other antibiotics were similar compared with the ATCC 17978 strain.

**Table 1:** **Susceptibility of** ***A. baumannii*** **ATCC 17978 selected with imipenem**

Antibiotics	17978	IPM-2 m	IPM-8 m
	Imipenem-selected concentration (mg/L)		
	0	0.5	2
Imipenem^a^	≦0.25	**1**	**≧16**
Meropenem^a^	≦0.25	**2**	**≧16**
Ceftazidime	4	4	4
Cefepime	2	2	2
Amikacin	≦2	≦2	≦2
Gentamicin	≦1	≦1	≦1
Ciprofloxacin	≦0.25	≦0.25	≦0.25
Levofloxacin	0.25	0.25	0.25
Ampicillin/subactam	≦2	4	4
Trimethoprim/Sulfamethoxazole	160	160	160

^a^A more than fourfold induction is indicated in boldface.

### Determination of the transcriptomes of imipenem-selected mutants and the parental strains

The total RNA fractions purified from IPM-2 m, IPM-8 m and ATCC17978 strains were analyzed to determine the respective gene expression levels and identify differentially expressed genes. Three libraries were constructed and subjected to paired-end sequencing using HiSeq 2000 (Illumina). The reads were aligned against the chromosomes and plasmids of *A. baumannii* ATCC 17978. A total of 11,995,382, 11,933,930, and 12,036,770 paired reads with lengths of 90 bases × 2 were obtained for IPM-2 m, IPM-8 m, and ATCC 17978, respectively. Approximately 99% of the transcribed genes aligned in the *A. baumannii* ATCC 17978 genome database (NC_009085.1) were recorded.

**Figure 1:**
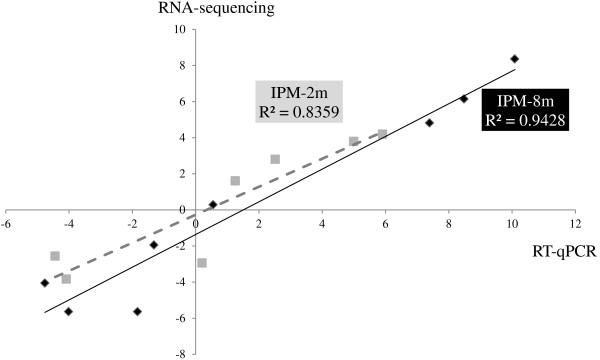
**Validation of the transcriptome results.** The transcriptomic results obtained through RNA sequencing were validated using qualitative RT-PCR (RT-qPCR) analysis. The level of differential expression of eight genes was compared, showing a correlation between RNA sequencing (Y-axis) and RT-qPCR analysis (X-axis). The level of differential expression between *A. baumannii* ATCC 17978 and their mutants is given as Log_2_-values. R^2^, the coefficient of determination.

The transcriptomic results, obtained using RNA sequencing, were validated through the RT-qPCR analysis of a subset of differentially expressed genes as shown in Figure 1. A good correlation was observed between the RT-qPCR data and the results obtained from the transcriptome analysis of IPM-2 m (R^2^ = 0.8359) and IPM-8 m (R^2^ = 0.9428).

### The gene expression profiles of imipenem-selected cells

**Figure 2:**
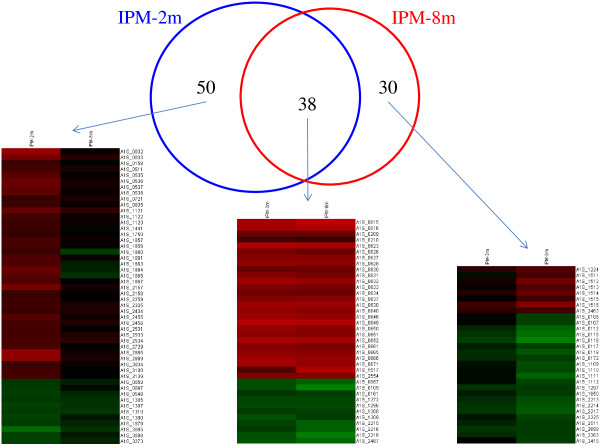
**The differentially expressed genes in IMP-2 m and IMP-8 m relative to the ATCC 17978 wild-type strain.** A Venn Diagram showing the relationship of differentially expressed genes between IPM-2 m and IPM-8 m. The heatmaps shown below demonstrate the expression patterns of the 50 genes unique to IPM-2 m, the 30 genes unique to IPM-8 m, and the 38 genes common to both strains.

The expression patterns of IPM-2 m vs. ATCC 17978 cells and IPM-8 m vs. ATCC 17978 cells were compared to identify differentially expressed transcripts. The up- and down-regulated genes were determined based on differences with *p* values below 0.05. Figure 2 shows the differentially expressed genes in IPM-2 m and IPM-8 m relative to the ATCC 17978 strain. A total of 88 and 68 genes were differentially expressed in IPM-2 m and IPM-8 m, respectively. Among these, 50 genes were highly expressed in IPM-2 m, 30 genes were highly expressed in IPM-8 m, and 38 genes were expressed common in both strains.

**Figure 3:**
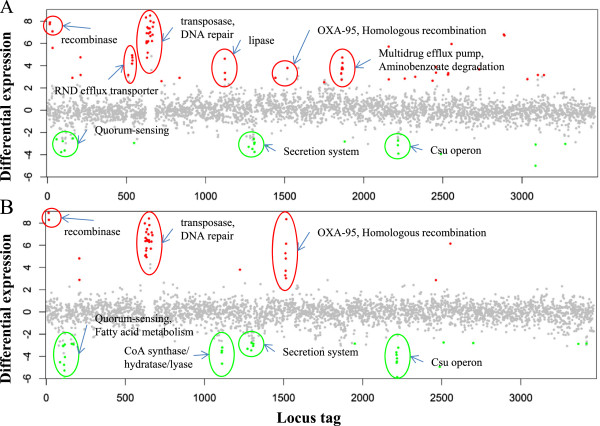
**Overview of the transcriptional difference between the IPM strains and the ATCC 17978 wild-type strain.** Comparative transcriptomics are displayed as differential expression (log_2_ transformed fold change) in **(A)** IPM-2 m and **(B)** IPM-8 m relative to ATCC 17978. The dots indicate the differential expression of all open reading frames, sorted on the *x*-axis according to the locus tag. Genes with p-values < 0.05 are considered differentially expressed. The red dots indicate up-regulated genes, whereas the green dots indicate down-regulated genes. Some differentially expressed genes are indicated in the literature and KEGG pathway information.

Figure 3 summarizes the transcriptional responses of ATCC 17978 upon selection with 0.5 mg/L (IPM-2 m) and 2 mg/L (IPM-8 m) imipenem. The differentially expressed genes were classified into functional groups based on COG category or KEGG pathways as shown in Table 2. Six groups of genes were identified: three groups were up-regulated, including recombinase, transposase and DNA repair, and beta-lactamase OXA-95 and homologous recombination, and three groups were down-regulated, including quorum sensing, secretion systems, and the csu operon, and these gene groups were simultaneously expressed in IPM-2 m and IPM-8 m mutants. In addition, three groups of genes, including the RND efflux pump, lipase, the multidrug efflux pump and aminobenzoate degradation, were up-regulated in IPM-2 m, and two groups of genes, including fatty acid metabolism and CoA synthase, hydratase and lyase, were down-regulated only in IPM-8 m. The genes with the highest overexpression were located in recombinase and transposase and DNA repair groups in IPM-2 m and IPM-8 m cells, highlighting the potential importance of these genes in carbapenem resistance in *A. baumannii.* Moreover, a rapid increase in *bla*_OXA-95_ (A1S_1517) expression from 14-fold (IPM-2 m) to 330-fold (IPM-8 m) suggests that *bla*_OXA-95_ might participate in carbapenem resistance. The rapid reduction gene expression upon imipenem induction was observed in the following groups: homoserine lactone synthase (A1S_0109), 17- (IPM-2 m) to 70-fold (IPM-8 m) reduction; quorum sensing group (A1S_0112, 0115, 0116,0117 and 0118), average 5- to 20-fold reduction; CoA synthase, hydratase and lyase group (A1S_1109, 1110 and 1111 ), 1.5- to 17-fold reduction; and the csu operon (A1S_2213 to A1S_2218), 8- to 25-fold reduction. Notably, many up-regulated genes were only restricted to IPM-2 m. Among 50 up-regulated genes, ten genes annotated as putative signal peptides were highly expressed in IPM-2 m cells, followed by a decrease in expression in IPM-8 m cells.

**Table 2:** **Functional groups of differentially expressed genes between mutant strains and ATCC 17978**

Locus tag	log2 (fold change)	log2 (fold change)	Protein name
	in IPM-2 m	in IPM-8 m	
**Recombinase**			
A1S_0015	7.74	8.92	Hypothetical protein A1S_0015
A1S_0016	7.90	8.30	Site-specific tyrosine recombinase
**Fatty acid metabolism**			
A1S_0106	-0.23	-3.05	Putative enoyl-CoA hydratase/isomerase
A1S_0107	-0.27	-3.08	Putative enoyl-CoA hydratase/isomerase family protein
A1S_0109	-3.62	-6.03	Homoserine lactone synthase
**Quorum sensing**			
A1S_0112	-1.86	-4.76	Acyl-CoA synthetase/AMP-acid ligases II
A1S_0115	-2.47	-5.27	Amino acid adenylation
A1S_0116	-2.97	-6.01	RND superfamily transporter
A1S_0117	-1.38	-2.92	Hypothetical proteinA1S_0117
A1S_0118	-2.01	-4.06	Hypothetical protein A1S_0118
**Transposase**			
A1S_0209	4.76	4.82	Transposase
A1S_0210	3.17	2.89	Transposase
**RND eflux transporter**			
A1S_0535	4.19	0.28	RND efflux transporter
A1S_0536	4.93	0.86	Macrolide transport protein
A1S_0537	4.74	0.83	RND efflux transporter
A1S_0538	4.48	0.39	RND efflux transporter
**Transposase and DNA repair**			
A1S_0623	8.34	7.98	DNA mismatch repair enzyme
A1S_0626	6.04	5.06	Hypothetical protein A1S_0626
A1S_0627	6.20	6.38	Hypothetical protein A1S_0627
A1S_0628	6.93	6.67	Putative transposase
A1S_0630	4.72	5.14	Hypothetical protein A1S_0630
A1S_0631	6.12	5.52	Hypothetical protein A1S_0631
A1S_0632	7.45	7.19	DNA primase
A1S_0633	6.13	5.74	Hypothetical protein A1S_0633
A1S_0634	5.17	4.93	Hypothetical protein A1S_0634
A1S_0637	6.21	6.52	DNA-directed DNA polymerase
A1S_0638	7.37	7.45	Hypothetical protein A1S_0638
A1S_0640	6.91	6.41	Hypothetical protein A1S_0640
A1S_0646	7.36	7.00	IcmB protein
A1S_0649	8.52	8.43	Putative phage primase
A1S_0650	7.02	6.46	Conjugal transfer protein
A1S_0651	6.67	6.32	TraB protein
A1S_0652	8.02	7.83	Putative ferrous iron transport protein A
A1S_0661	6.22	5.70	Phage integrase family protein
A1S_0665	6.79	6.34	Conjugal transfer protein TrbJ
A1S_0666	7.50	7.16	TrbL/VirB6 plasmid conjugal transfer protein
A1S_0671	7.89	6.92	Protein tyrosine phosphatase
**CoA synthase/hydratase/lyase**			
A1S_1109	-0.77	-3.66	Feruloyl-CoA synthase
A1S_1110	-0.48	-3.48	Hydroxybenzaldehyde dehydrogenase
A1S_1111	-0.41	-4.66	P-hydroxycinnamoyl CoA hydratase/lyase
A1S_1112	-0.53	-3.19	Putative 3-hydroxyphenylpropionic transporter MhpT
**Lipase**			
A1S_1121	4.63	2.24	Lipase/esterase
A1S_1122	3.36	0.67	Putative short-chain dehydrogenase
A1S_1123	2.77	0.59	Putative flavin-binding monooxygenase
**Bacterial secretion system, OOP family**			
A1S_1272	-3.30	-3.31	Putative transcriptional regulator
A1S_1296	-3.49	-3.45	Hypothetical protein A1S_1296
A1S_129	-2.42	-2.77	Hypothetical protein A1S_1297
A1S_1305	-2.59	-2.36	Putative outer membrane lipoprotein
A1S_1307	-3.03	-2.35	Putative ClpA/B-type chaperone
A1S_1308	-2.93	-3.06	Hypothetical protein A1S_1308
A1S_1309	-3.75	-2.86	Hypothetical protein A1S_1309
A1S_1310	-2.91	-2.58	Hypothetical protein A1S_1310
**Homologous recombination; Biosynthesis of secondary metabolites**			
A1S_1511	-0.58	3.30	Biotin synthase
A1S_1512	0.96	5.28	Putative ferredoxin
A1S_1513	0.27	3.71	Hypothetical protein A1S_1513
A1S_1514	1.61	4.81	Holliday junction nuclease
A1S_1515	0.24	3.04	Hypothetical protein A1S_1515
A1S_1516	2.80	6.15	Putative antibiotic resistance
A1S_1517	3.79	8.36	Beta-lactamase OXA-95
**Multidrug efflux pump; Aminobenzoate degradation**			
A1S_1750	2.52	0.26	AdeB
A1S_1857	3.29	0.51	Vanillate O-demethylase oxidoreductase
A1S_1858	3.32	0.36	Short-chain dehydrogenase/reductase SDR
A1S_1860	2.74	-2.86	Ring hydroxylating dioxygenase Rieske (2Fe-2S) protein
A1S_1861	3.70	0.53	Benzoate dioxygenase large subunit
A1S_1863	3.84	-1.41	Hypothetical protein A1S_1863
A1S_1864	4.73	-1.58	Acyl-CoA dehydrogenase-like protein
A1S_1865	4.25	-2.30	Glu-tRNA amidotransferase
A1S_1867	3.71	0.03	Major facilitator transporter
**Csu Operon**			
A1S_2213	-2.06	-3.63	CsuE
A1S_2214	-2.40	-3.86	CsuD
A1S_2215	-3.14	-4.57	CsuC
A1S_2216	-2.73	-4.45	CsuB
A1S_2217	-2.53	-4.18	CsuA
A1S_2218	-3.89	-5.88	CsuA/B
**ABC transporters**			
A1S_2531	3.20	0.22	Sulfate transport protein
A1S_2533	3.21	1.45	Putative esterase
A1S_2534	3.34	2.02	Sulfate transport protein
**Others**			
A1S_0032	7.09	0.49	Putative signal peptide
A1S_0033	5.60	1.73	Putative signal peptide
A1S_0058	-2.62	-1.47	Glycosyltransferase
A1S_0087	-3.76	-4.52	Short-chain dehydrogenase/reductase SDR
A1S_0097	-2.79	-0.41	Hypothetical protein A1S_0097
A1S_0158	2.91	-0.15	Hypothetical protein A1S_0158
A1S_0161	-2.53	-2.86	MFS family transporter
A1S_0172	-2.10	-2.92	Hypothetical protein A1S_0172
A1S_0511	3.17	1.25	Hypothetical protein A1S_0511
A1S_0548	-2.94	-1.92	TetR family transcriptional regulator
A1S_0721	2.62	1.15	Glutaryl-CoA dehydrogenase
A1S_0835	2.91	0.52	Outer-membrane lipoprotein precursor
A1S_1224	2.03	3.81	Transposase
A1S_1390	-3.20	-0.97	Hypothetical protein A1S_1390
A1S_1441	2.91	-0.31	Putative signal peptide
A1S_1879	-2.81	-1.76	Hypothetical protein A1S_1879
A1S_1950	-1.32	-2.85	Putative universal stress protein
A1S_2157	5.72	1.02	Putative signal peptide
A1S_2158	2.78	0.36	Putative monooxygenase
A1S_2225	-1.04	-3.21	Hypothetical protein A1S_2225
A1S_2259	2.84	0.15	Putative signal peptide
A1S_2325	3.00	1.40	Putative outer membrane protein
A1S_2434	2.65	1.60	Putative signal peptide
A1S_2455	3.89	0.89	Putative signal peptide
A1S_2456	3.38	1.59	LysR family transcriptional regulator
A1S_2463	2.16	2.87	Putative ribosomal large subunit pseudouridine synthase A(RluA-like)
A1S_2487	-3.93	-4.93	Hypothetical protein A1S_2487
A1S_2511	-1.62	-2.74	Phenylacetic acid degradation-related protein
A1S_2554	5.96	6.16	Putative transposase
A1S_2699	-2.51	-2.80	Putative transcriptional regulator
A1S_2729	3.67	-0.15	Outer-membrane lipoproteins carrier protein
A1S_2885	6.80	0.78	Putative signal peptide
A1S_2889	6.72	1.25	Putative signal peptide
A1S_3034	2.80	0.44	Hypothetical protein A1S_3034
A1S_3085	-5.00	-2.34	Putative flavohemoprotein
A1S_3086	-3.09	-1.21	Hypothetical protein A1S_3086
A1S_3100	3.18	0.18	Putative toluene tolerance protein (Ttg2D)
A1S_3139	3.16	0.03	Putative signal peptide
A1S_3273	-3.03	-1.79	Putative peptide signal
A1S_3363	-1.98	-2.86	Membrane metalloendopeptidases proteins
A1S_3415	0.09	-2.84	Maleylacetoacetate isomerase

Table 3 shows the comparative results of differentially expressed genes in imipenem-selected mutants and biofilm-associated ATCC 17978, as previously described [11]. Many biofilm-associated genes, including quorum sensing-associated genes (A1S_0109, A1S_0112 and A1S_0115) and the CsuAB-A-B-C-D-E chaperone-usher secretion system (A1S_2214, A1S_2215 and A1S_2218), were inversely expressed in imipenem-selected mutants. However, four genes encoding the RND efflux transporter, sulfate transport protein and putative signal peptides, were overexpressed in both strains, indicating that those genes might participate in pathways overlapping carbapenem resistance and biofilm formation.

**Table 3:** **Comparison of differentially expressed genes between imipenem-selected mutants (this study) and biofilm-associated ATCC 17978 cells as decribed by Rumbo-Feal** ***et al.*** **(12)**

Locus tag	Log2 (fold change) in IPM-2 m	Log2 (fold change) in IPM-8 m	Log2 (fold change) biofim vs.expotenetial phase cells ^a^	Protein Name
**Inverse expressed between imipenem-resistant mutants and biofilm-associated cells**				
NameA1S_0087	-3.76	-4.52	1.36	Short-chain dehydrogenase/reductase SDR
A1S_0109	-3.62	-6.03	5.91	Homoserine lactone synthase
A1S_0112	-1.86	-4.76	6.23	Acyl-CoA synthetase/AMP-acid ligases II
A1S_0115	-2.47	-5.27	7.24	Amino acid adenylation
A1S_0116	-2.97	-6.01	5.81	RND superfamily transporter
A1S_0117	-1.38	-2.92	4.58	Hypothetical protein A1S_0117
A1S_0118	-2.01	-4.06	3.21	Hypothetical protein A1S_0118
A1S_2214	-2.40	-3.86	7.49	CsuD
A1S_2215	-3.14	-4.57	7.65	CsuC
A1S_2218	-3.89	-5.88	7.36	CsuA/B
**Overexpressed both in imipenem-resistant mutants and biofilm-associated cells**				
A1S_0538	4.48	0.39	2.72	RND efflux transporter
A1S_2534	3.34	2.02	4.40	Sulfate transport protein
A1S_0032	7.09	0.49	5.01	Putative signal peptide
A1S_2889	6.72	1.25	5.54	Putative signal peptide

### Measurement of carbapenemase hydrolysis

**Figure 4:**
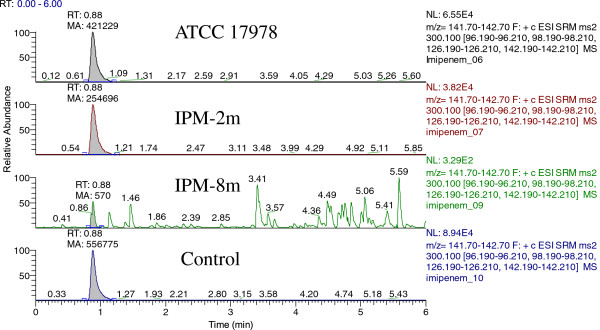
**LC-MS/MS chromatogram of imipenem under co-incubating with** ***A. baumannii*** **ATCC 17978, IPM-2 m and IPM-8 m cells.** Control, imipenem standard solution.

To examine carbapenemase hydrolysis in ATCC 17978, IPM-2 m and IPM-8 m cells, LC-MS/MS was performed, and the results are shown in Figure 4. The rate of imipenem hydrolysis was calculated by dividing the imipenem area after the incubation procedure by *A. baumannii* ATCC 17978 area. Compared with IPM-2 m, the rate of imipenem hydrolysis in IPM-8 m showed a 430-fold increase.

### Quantitative analysis of biofilm formation

**Figure 5:**
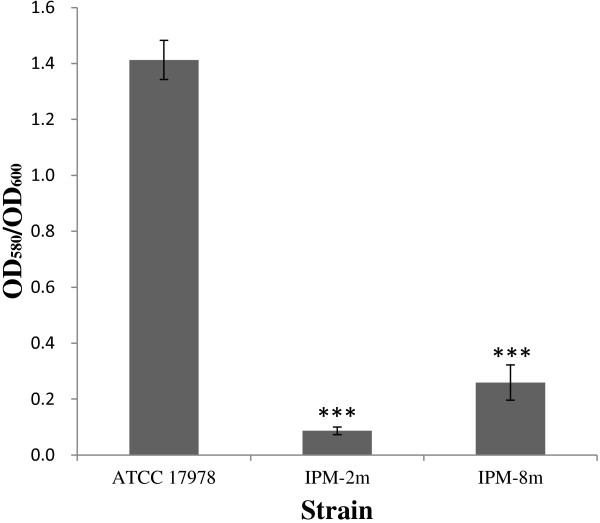
**Quantification of biofilm formation in** ***A. baumannii*** **strains on plastic surface.** To determine total cell mass the OD_600_ was measured after the cultures were briefly sonicated to resuspend most of the cells. The OD_580_ was measured after the stained tubes were incubated with ethanol-acetone. The error bar show the S.D. ***,*p* < 0.0001 using Student’s *t*-test comparing mutant and wild-type strains.

To clarify the association between biofilm formation and carbapenem resistance in *A. baumannii*, biofilm formation in ATCC 17978 and imipenem-selected mutants was quantitative analyzed as shown in Figure 5. A significant decrease in biofilm formation (p < 0.001) was observed in IPM-2 m and IPM-8 m cells, indicating an inverse relationship between carbapenem resistance and biofilm production in *A. baumannii* ATCC 17978.

## Discussion

In the present study, we successfully constructed an antibiotic-induction platform to observe dynamic transcriptome changes upon carbapenem selection. *A. baumannii* ATCC 17978 was selected as the study material based on three advantages. First, the complete genome of this organism has been sequenced since 2007 [15]. Second, the MICs for most commonly used antibiotics, such as the 3rd cephalosporins, aminoglycosides, carbapenems and fluoroquinolones, are still susceptible; thus, *A. baumannii* ATCC 17978 would be a model candidate for antibiotic selection experiments. Third, the gradual increase of MIC observed only for carbapenem suggests that the carbapenem-specific resistance mechanism could be studied using an imipenem-selected platform.

In our previous study using genome-wide analysis [14], we demonstrated that imipenem exposure at a concentration of 0.5 mg/L only mediated the transposition of IS*Aba1* upstream of the *bla*_OXA-95_ gene. None of the other genes in imipenem-selected mutants were modified, rearranged or acquired by horizontal-gene transfer upon imipenem exposure compared to their parental strains. To continue the previous study, herein, we examined the transcriptional profiles of *A. baumannii* ATCC 17978 upon selection with imipenem gradient. Despite all the gene expression analysis were done with strains cultured in the absence of antibiotics, the MICs to imipenem in IPM-2 m and IPM-8 m remained unchangeable, indicating that these mutants were stable. Besides, the MICs observed in response to imipenem selection in IPM-2 m and IPM-8 m cells were 1 and >16 mg/L, respectively, reflecting imipenem-susceptibility and imipenem-resistance according to the CLSI guidelines [16]. Thus, the results of the present study showed the dynamic changes in the transcriptome profiles, from imipenem-susceptible to imipenem-resistance during the selection period, representing the first study to demonstrate the potential mechanisms underlying carbapenem resistance in *A. baumannii* ATCC 17978.

Thus, several novel findings have been revealed in the present study. First, many of the highly expressed genes encoding proteins for recombinase, transposase, and DNA repair were simultaneously observed in IPM-2 m and IPM-8 m cells. This result suggests that genome recombination might play an important role in conferring carbapenem resistance, consistent with the conclusions of several reports using genetic analysis [17, 18]. Second, the overexpression of several genes involved in RND efflux transporters and fatty acid metabolism were observed in IPM-2 m cells, and this expression was reduced in IPM-8 m cells. Despite several reports emphasizing the major role of efflux pumps in the development of antimicrobial resistance in *Acinetobacter* spp. [19, 20], the results obtained herein are consistent with those of previous reports showing that efflux pumps, particularly RND-type transporters, play an important role in the initial exposure to imipenem and are subsequently down-regulated during carbapenem resistance. In other words, efflux pumps alone may be not sufficient to provide protection against a high concentration of carbapenem. Third, several genes involved in quorum sensing and the CsuAB-A-B-C-D-E chaperone-usher secretion system are down-regulated upon selection with imipenem. The disruption of these genes has been associated with a decrease in biofilm formation [11]. The results of other studies concerning carbapenem resistance and biofilm formation showed reduced biofilm formation in meropenem (MEM)-resistant *A. baumannii* isolates compared with MEM-susceptible strains [21], consistent with the results obtained in either biofilm-associated gene expression or the phenotypic determination of biofilm production in *A. baumannii* strains. To date, the ability of *A. baumannii* to form biofilms that adhere to and persist on a broad range of surfaces might be key to revealing the pathogenic mechanisms of this microorganism [22]. Therefore, we hypothesize that carbapenem resistance might reduce virulence through the reduction of biofilm production in some *A. baumannii* strains.

The rapid adaption to the environment might emphasize the ability of microorganisms to live under external stress. Dynamic changes in genome architecture and gene expression are required for organisms to survive in their environment. Dynamic changes in the gene expression of *A. baumannii* have been observed in biofilm compared with planktonic cells using whole transcriptome analysis [11]. Also, the transcriptional responses of *A. baumannii* to environmental stress have been reported [23, 24]. For example, several siderophore biosynthesis genes were up-regulated in response to iron starvation and therefore are likely to be important for the survival of *A. baumannii* in iron-limited environments. In addition, various type IV pilus genes were also down-regulated [23]. In the present study, dynamic changes in the transcriptional responses to carbapenem concentrations ranging from 0.5 mg/L (mild stress) to 2 mg/L (stringent stress) have also been observed. Herein, we propose a "bacterial energy conversion hypothesis" to describe the dynamic changes in the transcriptome upon carbapenem stress in *A. baumannii* ATCC 17978. First, the net energy required for metabolism is constant throughout the life of the cell. For rapid adaption and survival upon environmental stress, many genes in cells are monitored and up-regulated to overcome the external stress, and much energy is required for the expression of these genes. However, several genes that are not required for survival are down-regulated to save energy. In the present study, several genes, including the RND efflux transporter, lipase, recombination-associated proteins, and *bla*_OXA-95_, are up-regulated in *A. baumannii* upon exposure to mild carbapenem stress. Several biofilm-associated genes, including quorum sensing, protein secretion system and the CsuAB-A-B-C-D-E chaperone-usher secretion system, which could be not required against for imipenem pressure, have been down-regulated. To adapt to a more stringent environment, the overexpression of target survival genes, e.g. *bla*_OXA-95_, is needed, resulting in the consumption of most of the energy in the cell. Thus, some of the genes up-regulated during mild stress, e.g. efflux pumps, are down-regulated so as to transform excess energy and maintain cell viability despite efflux pumps play important roles in the resistance to antibiotics [25]. The bacterial energy conversion hypothesis requires more evidences to verify, however, in the present study, the results of transcriptomic analysis and LC-MS/MS demonstrated that *bla*_OXA-95_ might play a critical role in survival upon exposure to stringent carbapenem stress in *A. baumannii* ATCC 17978. Moreover, the transposition of IS*Aba1* upstream of the *bla*_OXA-95_ gene is observed upon exposure to mild carbapenem stress, suggesting that the upstream signaling pathway linking external stress and IS*Aba1* transposition may be a critical mechanism for carbapenem resistance in *A. baumannii* ATCC 17978.

## Conclusions

This study defined the global transcriptional response of *A. baumannii* to imipenem exposure. The up-regulation of recombination-associated genes and *bla*_OXA-95_ was the predominant feature of this transcriptional response. Several genes involved in biofilm formation, such as quorum sensing, protein secretion system and the CsuAB-A-B-C-D-E chaperone-usher secretion system, were down-regulated upon imipenem selection, resulting in the reduction of biofilm production. Overall, the results indicated that *A. baumannii* adapts to an environment with carbapenem availability.

## Methods

### Bacterial strains

*A. baumannii* ATCC 17978 was used as a parental strain. The carbapenem-selected mutants were generated from the parental strain using a previously described method [26]. The selected strains exposed to 0.5 and 2 mg/L imipenem were collected during the induction period and referred to as IPM-2 m and IPM-8 m. The genotypic patterns in the selected mutants and the parental strain were determined using PFGE, as previously described [27].

### Antimicrobial susceptibility

The susceptibility of the *Acinetobacte*r mutants and the parental strain to antimicrobial agents was determined using a microdilution method in accordance with the guidelines of the Clinical and Laboratory Standards Institute [16]. The agents tested included ampicillin/sulbactam, ceftazidime, cefepime, amikacin, gentamicin, ciprofloxacin, levofloxacin, trimethoprim/sulfamethoxazole, imipenem and meropenem. *Escherichia coli* strain ATCC 25922 and *Pseudomonas aeruginosa* strain ATCC 27853 were used as reference controls for the susceptibility testing. A four-fold or greater induction in the minimum inhibitory concentration (MIC) values after exposure to imipenem was considered significantly different from the control.

### RNA isolation and library preparation for transcriptome sequencing

*A. baumannii* cultures were grown to log phase (OD_600_ 1.00) in Muller Hinton broth with shaking at 37°C before RNA extraction. Total RNA was isolated from cells using the PureLink™ Micro-to-Midi Total RNA Purification System (Invitrogen, Life Technologies, Carlsbad, CA, USA) according to the manufacturer’s instructions. The RNA quantity and quality were assessed using a BIOANALYZER 2100 (Agilent Technologies Inc., Germany), followed by RNA-Seq. The RNA integrity number (RIN) of total RNA should be greater than 8.0, and rRNA ratio (23S/16S) should be greater than 1.2.

The RNA-sequencing library was prepared as previously described [28]. The constructed sequencing libraries were sequenced using the Illumina HiSeq 2000 platform at Beijing Genome Institute (BGI, Shenzhen, China).

### Analysis of the RNA-Seq data

The sequenced libraries were mapped against predicted transcripts from the *Acinetobacter baumannii* ATCC 17978 genome using TopHat v2.0.4 [29]. The transcript abundance (FPKM, Fragments Per Kilobase of exon per Million fragments mapped) and significant changes in transcript expression were estimated using Cufflinks v2.0.2 [30, 31]. Transcripts with *p*-values less than 0.05, determined using CuffDiff [31], were considered differentially expressed between mutant and wild-type strains. The transcripts were annotated with Cluster of Orthologous Groups (COG) and protein functions according to their locus tags [32]. The functional groups comprising differentially expressed transcripts were manually curetted based on COG annotation, Kyoto Encyclopedia of Genes and Genomes (KEGG) [33], and studies cited in the corresponding main text. Raw sequences were deposited at the NCBI sequence Read Archive under the Bioproject accession number PRJNA244702.

### Quantitative biofilm formation

Biofilm formation on polystyrene was assessed through the crystal violet staining of cells cultured in LB broth as previously described [34]. Each experiment was performed in triplicate and repeated three times.

### Reverse transcriptase-quantitative PCR (RT-qPCR)

Gene expression was analyzed using a previously described method [26]. Briefly, total RNA was isolated from 1 × 10^9^*A. baumannii* cells. After DNase treatment of the RNA samples and cDNA synthesis, RT-qPCR was performed as previously described [26]. The template cDNA was diluted 1:100, and 2.5 μl was added to SYBR green PCR master mix (Biogenesis Technologies, Inc., Taiwan) for each reaction. An Eco Real-Time PCR System (Illumina) was used for analysis. Internal forward and reverse primers for each gene were designed using the DesignStudio web-based tool (Illumina), as described in supporting information (Additional file 1: Table S1). The experiments were repeated in triplicate independent experiments. Normalization to the 16S ribosomal gene facilitated the calculation of the fold-changes using the threshold cycle (*CT*) method [35].

### Detection of carbapenemase hydrolysis using LC-MS/MS

Each strain was analyzed using LC-MS/MS to detect imipenem hydrolysis as previously described [36]. Briefly, the strains were cultured overnight on Mueller-Hinton agar. The bacteria were dissolved in normal saline solution and adjusted to OD_600_ = 2.0. A 1-mL volume of this suspension was incubated with 5 μg/mL of imipenem for 1 h at 37°C with smooth agitation. The suspensions were subsequently centrifuged at 12,000 g for 5 min, and 300 μL of supernatant was mixed with 700 μL of methanol. After centrifugation at 12,000 g for 5 min, 200 μL of supernatant was mixed with 800 μL of water. The abundance of imipenem was measured through LC-MS/MS using the Thermo Accela LC system (Waltham, MA) coupled to a TSQ Quantum tandem triple-quadrupole mass spectrometer. Briefly, the chromatography step was performed using a fused-core Poroshell C_18_ column (Agilent) and eluted with mobile phase A (0.1% formic acid in water) and B (0.1% formic acid in methanol). Chromatographic separation was achieved through gradient elution at a flow rate of 0.32 mL/min. The injection volume was 10 μL. The retention time for imipenem was 0.88 min. Ionization was achieved using electrospray in positive ionization mode (ESI^+^). The multiple-reaction-monitored parameters were optimized through post-column infusion of the stock solution (1 μg/mL) using Quantum TuneMaster software (ThermoFisher).The parameters included tube lens (72.3) and collision energy (27 V) for imipenem transition (*m/z* 300.1 > 142.2).

### Statistical analysis

The differences in biofilm production between imipenem-selected mutants and the parental strains were analyzed using Student’s t-test, as appropriate. The differences between the two groups of isolates were considered significant at the p < 0.05 level. The data entry and analyses were performed using the Statistical Package for the Social Sciences (SPSS) software version 15.0 (SPSS Inc., Chicago, IL, USA).

## Electronic supplementary material

Additional file 1: Table S1: Oligonucleotides used in RT-qPCR. (XLSX 10 KB)
